# A Machine Learning Approach for Mortality Prediction in COVID-19 Pneumonia: Development and Evaluation of the Piacenza Score

**DOI:** 10.2196/29058

**Published:** 2021-05-31

**Authors:** Geza Halasz, Michela Sperti, Matteo Villani, Umberto Michelucci, Piergiuseppe Agostoni, Andrea Biagi, Luca Rossi, Andrea Botti, Chiara Mari, Marco Maccarini, Filippo Pura, Loris Roveda, Alessia Nardecchia, Emanuele Mottola, Massimo Nolli, Elisabetta Salvioni, Massimo Mapelli, Marco Agostino Deriu, Dario Piga, Massimo Piepoli

**Affiliations:** 1 Department of Cardiology Guglielmo Da Saliceto Hospital Piacenza Italy; 2 PolitoBIOMed Lab Department of Mechanical and Aerospace Engineering Politecnico di Torino Torino Italy; 3 Anesthesiology and ICU Department Guglielmo da Saliceto Hospital Piacenza Italy; 4 TOELT LLC - AI Research and Development Dubendorf Switzerland; 5 Department of Clinical Sciences and Community Health Centro Cardiologico Monzino Istituto di Ricovero e Cura a Carattere Scientifico Milano Italy; 6 Department of Clinical and Experimental Medicine University of Parma Parma Italy; 7 Dalle Molle Institute for Artificial Intelligence Università della Svizzera italiana/Scuola universitaria professionale della Svizzera italiana Lugano Switzerland; 8 Istituto Istruzione Superiore Casalpusterlengo Italy; 9 7HC SRL Rome Italy

**Keywords:** artificial intelligence, prognostic score, COVID-19, pneumonia, mortality, prediction, machine learning, modeling

## Abstract

**Background:**

Several models have been developed to predict mortality in patients with COVID-19 pneumonia, but only a few have demonstrated enough discriminatory capacity. Machine learning algorithms represent a novel approach for the data-driven prediction of clinical outcomes with advantages over statistical modeling.

**Objective:**

We aimed to develop a machine learning–based score—the Piacenza score—for 30-day mortality prediction in patients with COVID-19 pneumonia.

**Methods:**

The study comprised 852 patients with COVID-19 pneumonia, admitted to the Guglielmo da Saliceto Hospital in Italy from February to November 2020. Patients’ medical history, demographics, and clinical data were collected using an electronic health record. The overall patient data set was randomly split into derivation and test cohorts. The score was obtained through the naïve Bayes classifier and externally validated on 86 patients admitted to Centro Cardiologico Monzino (Italy) in February 2020. Using a forward-search algorithm, 6 features were identified: age, mean corpuscular hemoglobin concentration, PaO_2_/FiO_2_ ratio, temperature, previous stroke, and gender. The Brier index was used to evaluate the ability of the machine learning model to stratify and predict the observed outcomes. A user-friendly website was designed and developed to enable fast and easy use of the tool by physicians. Regarding the customization properties of the Piacenza score, we added a tailored version of the algorithm to the website, which enables an optimized computation of the mortality risk score for a patient when some of the variables used by the Piacenza score are not available. In this case, the naïve Bayes classifier is retrained over the same derivation cohort but using a different set of patient characteristics. We also compared the Piacenza score with the 4C score and with a naïve Bayes algorithm with 14 features chosen a priori.

**Results:**

The Piacenza score exhibited an area under the receiver operating characteristic curve (AUC) of 0.78 (95% CI 0.74-0.84, Brier score=0.19) in the internal validation cohort and 0.79 (95% CI 0.68-0.89, Brier score=0.16) in the external validation cohort, showing a comparable accuracy with respect to the 4C score and to the naïve Bayes model with a priori chosen features; this achieved an AUC of 0.78 (95% CI 0.73-0.83, Brier score=0.26) and 0.80 (95% CI 0.75-0.86, Brier score=0.17), respectively.

**Conclusions:**

Our findings demonstrated that a customizable machine learning–based score with a purely data-driven selection of features is feasible and effective for the prediction of mortality among patients with COVID-19 pneumonia.

## Introduction

Despite measureless efforts to limit the spread of COVID-19, over 100 million people have been confirmed positive for SARS-CoV-2 infection and more than 2 million people have died from the virus worldwide, as of February 10, 2021 [1]. While these numbers are rapidly increasing day by day, hospitals have been receiving requests beyond capacity and face extreme challenges concerning a sharp increase in the demand for medical resources as well as a shortage of hospital beds and critical care equipment for the timely treatment of ill patients. Additionally, the clinical spectrum of SARS-CoV-2 infections ranges from asymptomatic status to severe viral pneumonia with respiratory failure and even death, making reliable and successful patient triaging challenging [2].

Data from epidemiological studies suggest that severe illness occurs in approximately 20% of patients and that older age, coexisting medical conditions, and cardiovascular risk factors are associated with worse prognosis [3,4]. In this scenario, identification of the key patient variables driving COVID-19 prognosis is of paramount importance to assist physicians in the early prediction of the pathology trajectory and to improve patient outcomes.

To date, several prognostic models combining clinical and laboratory parameters have been proposed, but they included mainly patients from the first wave of the COVID-19 pandemic. This may cause a risk of bias, making these models unsuitable for clinical decision in daily practice [5,6].

The increasing use of electronic health record (EHR) systems has increased the availability of a large amount of data suitable for machine learning analysis. The latter has already proven its potential to support clinical decisions in many medical fields, including the COVID-19 pandemic [7,8]. Therefore, the aim of this study was to develop and validate a new scoring technique—the Piacenza score—to predict the prognosis of COVID-19 pneumonia, based on a machine learning technique with a purely data-driven selection of prognostic features collected at hospital admission.

We hypothesized that a machine learning score based on data-driven selection of features, which is different from inference statistics, could capture nonlinear relationships among clinical features without human-biased intervention and predict mortality for individual patients more accurately than the currently available risk scores.

## Methods

### Population and Collected Data

The study was conducted at Guglielmo da Saliceto Hospital, which serves a population of about 300,000 people in the area of Piacenza, Emilia Romagna, in northern Italy. This region has the second highest number of COVID-19 deaths in the country (6219 as of December 7, 2020).

This study retrospectively analyzed the EHRs of a cohort of 852 patients diagnosed with COVID-19 pneumonia according to the World Health Organization interim guidance and admitted to the hospital from February to November 2020. COVID-19 infection was diagnosed by a positive result on a reverse transcriptase–polymerase chain reaction (RT-PCR) assay of a specimen collected on a nasopharyngeal swab. Pregnant women, children (<18 years), and patients with a negative RT-PCR assay were excluded from the study as well as patients presenting with shock and coma.

Data collected in the EHR included patients’ demographic information, comorbidities, triage vitals, and laboratory tests and outcomes (including length of stay, discharge, readmission, and mortality). Routine blood examinations at admission comprised complete blood count, coagulation profile, and serum biochemical tests (including renal and liver function, creatine kinase, lactate dehydrogenase, electrolytes, and C-reactive protein). A total of 62 patient characteristics were considered in the score design and development. The study protocol was approved by the local committee on human research.

### Criteria for Discharge and Outcome

The criteria for discharge were at the discretion of the caregiver physician. In most cases, the criteria encompassed absence of fever for at least 3 days, as well as substantial clinical improvement including clinical remission of symptoms and 2 throat-swab samples negative for SARS-CoV-2 RNA obtained at least 24 hours apart. The primary outcome was 30-day in-hospital mortality.

### Piacenza Score Design

The Piacenza score is a machine learning–based COVID-19 mortality risk predictor. It was implemented using a naïve Bayes approach, which is a probabilistic classifier describing the dependence from the outcome of each variable characterizing the patient, taken separately from the others. The naïve Bayes algorithm was chosen due to the following advantages: (1) it provides a probability of the final outcome, which thus represents the mortality risk; (2) it can handle both categorical and continuous features; and (3) it can handle missing values, thus providing a mortality risk even when all variable inputs for a patient are not available. Moreover, it proved a successful approach in predicting clinical outcomes in several medical scenarios [9,10]. Other key advantages of using a naïve Bayes classifier are its easy implementation, computational efficiency, optimal scaling performance, and the fact that it achieves good results even in small data sets. Furthermore, it is not influenced by irrelevant features or outliers.

Its major limitation stands in the assumption at the core of the method: features independence. Even if this assumption is almost never satisfied, the classifier proved to reach reasonable results in many scenarios, especially in text classification. Another drawback of naïve Bayes is that if a categorical feature presents a value in the test data set, which was not observed in the training data set, then the model will be unable to make a prediction. Nevertheless, this issue can be solved with various smoothing techniques. Patients missing some features can be easily handled. In fact, only the features’ probability distributions need to be computed in training a naïve Bayes classifier. Thus, no imputation was performed and all patients were included in the training phase, since not all missing data were considered for every feature. Furthermore, when applying the trained model to make inferences, the final user can insert missing data, still obtaining a reliable result.

### Derivation and Test Cohorts

The EHRs of 852 patients were randomly split into derivation (70%) and test (30%) cohorts. The derivation cohort was first used to select, among the considered 62 patient features, the most significant ones, and then to train the naïve Bayes classifier using only the best predictors, while the predictive ability of the estimated model was assessed on the test cohort.

### Piacenza Score Development, Optimization, and Identification of Variable Importance

The Piacenza score has been developed and tailored to (1) minimize the number of clinical variables to be ingested and (2) to maximize the overall prediction performance (ie, in terms of maximization of the area under the receiver operating characteristic curve [AUC]) and patient stratification ability. The most significant patient features were identified through the so-called forward-search approach [11].

The forward-search approach is a purely data-driven dimensionality reduction technique that is able to identify, given a large set of input features, the minimum combination of those features, which maximizes the performance metrics associated with a machine learning algorithm. The forward-search approach was employed here to reduce the number of patient variables from 62 to the 6 most relevant ones used to train the naïve Bayes classifier.

### Piacenza Score Evaluation and Metrics

The test cohort was used to assess the performance of the Piacenza score. In order to increase the statistical significance of the results, bootstrapping was used to randomly generate 100 test sets from the original test cohort. Moreover, an external validation cohort has been considered to further validate the Piacenza score performance. The external validation cohort consisted of data from 86 patients with COVID-19 enrolled at Centro Cardiologico Monzino Hospital (Milan, Italy).

The performance of Piacenza score was evaluated in terms of discrimination and calibration capabilities. The discrimination ability was determined by computing the receiver operating characteristic (ROC) curve on the test cohort and the associated AUC, together with its 95% CI. As additional metrics, the negative predictive value (NPV), the positive predictive value (PPV), the accuracy, the sensitivity, the specificity, and the F1 and F2 scores were computed. These metrics were calculated for a threshold value obtained by maximizing the F2 score. The calibration ability was derived by the so-called calibration plots, which compare observed and predicted outcomes with associated uncertainties. The Brier index was used to evaluate the ability of machine learning to stratify and predict observed outcomes. The Brier index is defined as the mean-squared difference between the observed and predicted outcomes and ranges from 0 to 1, with 0 representing the best calibration.

Finally, the variable relative importance was quantified for the identified 6 most relevant patient features. The relative importance is a comparative measure of the patient feature’s weight in determining the Piacenza risk score.

### Usability, Flexibility, and Customization

The Piacenza score was specifically designed to be an easy, fast, versatile, fair, open, and user-friendly tool. To reach this goal, a web-based calculator of the score, via a website, was released [12]. This calculator can be used by clinicians to estimate a hospitalized patient’s risk of 30-day mortality.

We added a tailored version of the algorithm to the website, which enables an optimized computation of the mortality risk score for a patient even when some variables used by the Piacenza score are not available. In this case, the naïve Bayes classifier is retrained over the same derivation cohort but using a different set of patient characteristics. Moreover, a second naïve Bayes model has been presented as a possible example of the Piacenza score’s customization and flexibility. The above-mentioned model has been trained with the following 14 variables, chosen a priori by the physician for their association with mortality in COVID-19 pneumonia: age, gender, diabetes, length of symptoms before hospital admission, systolic blood pressure, respiratory rate, PaO_2_/FiO_2_ ratio, platelets and eosinophils count, neutrophil-to-lymphocyte ratio, C-reactive protein, direct bilirubin, creatinine, and lactate dehydrogenase. Finally, we compared the performance of the Piacenza score with the above-mentioned “clinical” naïve Bayes classifier to show the flexibility of the method, which can be easily retrained with another subset of predictors.

### Website Design and Development

The website has been developed in Python (Python Software Foundation), using the Flask framework, and Hosting is managed through Docker.

The site consists of three main pages: *Home*, *Custom Analysis*, and *Multiple Analysis*. The *Home* and the *Custom Analysis* pages require submitting a form that is dynamically composed in the backend through a Python dictionary variable. This allows us to easily change the form without changing the HTML code. The current dictionary contains the following fields characterizing the features: name, type (continuous or binary), measurement unit, information for the user, value, and mandatory flag.

On the *Home* page, once a form is submitted, the backend receives and sends the parsed data to the previously trained naïve Bayes classifier, which computes the mortality risk that is visualized on the website, typically in less than 1 second. On the *Custom Analysis* page, once a form is submitted and parsed, a naïve Bayes classifier is trained using only the specified features. Since the overall training process may be time-consuming as it also performs feature selection, the final results are automatically sent to the email address specified by the user after completing the training. The *Multiple Analysis* page allows users to compute mortality risks for many patients, without the need to manually fill in a form for every single patient. Clinicians are requested to submit a CSV (comma-separated values) file containing the values of the 6 features characterizing the Piacenza score. An example of the structure of a CSV file is provided on the website.

### Comparison With Conventional Risk Models

To further assess the performance of the Piacenza score, we compared it with the 4C mortality score, which considers the following predictors: age, gender, number of comorbidities, respiratory rate, peripheral oxygen saturation (sO_2_), level of consciousness (Glasgow coma scale), urea level, and C-reactive protein. The same test cohort used to test the Piacenza score was employed.

### Statistical Analysis

Categorical variables were reported as count (%) and continuous variables as mean (SD). A two-sided *P* value <.05 was considered statistically significant. We used the Fisher exact test to assess differences between binary variables and the Welch two-sample *t* test to assess differences between continuous variables. The overall implementation of all codes for the machine learning score and analysis tools was performed in the Python 3.7.4 environment. The Python libraries employed were *pandas* (for data set management), *NumPy* (for numerical computations), and *sklearn* (for data set preprocessing; eg, data set splitting). The naïve Bayes classifier at the core of the Piacenza score was manually implemented (without any additional machine learning framework used) since an existing algorithm for naïve Bayes classification dealing both with continuous and categorical variables as well as missing data was not available in the *sklearn* library. The forward-search algorithm for feature selection was also manually implemented.

## Results

### Patient Characteristics and Events

A total of 852 patients with SARS-CoV-2 pneumonia were hospitalized during the study period, of which 242 (28%) were admitted to the intensive care unit (ICU). The mean age of the patients was 70 (SD 14) years, and 599 (70%) were male. Comorbidities were present in 602 patients (71%): mainly arterial hypertension (n=499, 59%), dyslipidemia (n=205, 24%), and diabetes (n=157, 18%). The mean time between onset of symptoms and hospital admission was 6.5 (SD 3.9) days. Fever (n=776, 91%), dyspnea (n=543, 64%), and cough (n=400, 47%) were the most common symptoms at admission. A total of 293 patients (34%) died within 30 days after hospital admission. The median time from hospital admission to discharge or death was 9 days. A comparison of clinical characteristics between survivors and nonsurvivors showed that the latter were older *(P*<.001) and had a higher prevalence of hypertension and cerebrovascular disease (*P*<.001); longer symptom duration (*P*<.001); higher respiratory rate (*P*<.001); and lower SpO_2_ (*P*<.001), PaO_2_/FiO_2_ ratio (*P*<.001), and systolic blood pressure at admission (*P*=.02) (Table 1).

**Table 1:** Study population characteristics and a comparison of survivors and nonsurvivors.

Characteristic		All patients (N=852)	Patients discharged alive (n=559)	Deceased patients (n=293)	*P* value^a^	
Gender (male), n (%)		599 (70)	386 (69)	213 (73)	.30	
Age (years), mean (SD)		70 (14)	65 (14)	78 (10)	*.001*	
**Comorbidities, n (%)**		602 (71)	364 (65)	238 (81)	*.001*	
	Hypertension	499 (59)	294 (53)	205 (70)	*.001*	
	Atrial fibrillation	109 (13)	58 (10)	51 (17)	*.005*	
	Chronic obstructive pulmonary disease	130 (15)	76 (14)	54 (18)	.07	
	Dyslipidemia	205 (24)	132 (24)	73 (25)	.67	
	Chronic kidney disease	75 (9)	42 (8)	33 (11)	.07	
	Diabetes	157 (18)	90 (16)	67 (23)	*.02*	
	Cancer	65 (8)	38 (7)	27 (9)	.22	
	Stroke	28 (3)	9 (2)	19 (6)	*.001*	
	Peripheral artery disease	19 (2)	10 (2)	9 (3)	.23	
	Coronary artery disease	96 (11)	58 (10)	38 (13)	.26	
**Symptoms**						
	Time from symptom onset to admission, mean (SD)	6.54 (3.94)	6.71 (3.79)	6.27 (4.16)	*.001*	
	Fever, n (%)	776 (91)	513(92)	263(90)	.32	
	Dyspnea, n (%)	543 (64)	317(57)	225(77)	*.002*	
	Cough, n (%)	400 (47)	280 (50)	120 (41)	.18	
	Fatigue, n (%)	174 (20)	118 (21)	56 (19)	.32	
	Diarrhea, n (%)	77 (9)	66 (12)	11(4)	.05	
	Syncope, n (%)	43 (5)	36 (6.5)	7 (2)	.18	
**Baseline clinical findings, mean (SD)**						
	PaO_2_/FiO_2_ ratio	225.93 (96.34)	270.54 (83.82)	196.54 (92.70)	*.001*	
	pH	7.45 (0.07)	7.46 (0.07)	7.45 (0.07)	.35	
	PaO_2_	60.16 (18.58)	59.68 (15.94)	60.56 (20.54)	.71	
	PaCO_2_	35.75 (10.37)	35.36 (8.52)	36.05 (11.58)	.62	
	HCO_3_	25.43 (6.78)	26.22 (9.12)	24.81 (3.97)	.23	

^a^*P* value refers to either the Student *t* test or the chi-square test. Italicized values are significant.

Major laboratory markers were tracked upon admission. Specifically, lactate dehydrogenase, creatine kinase, cholinesterase, creatinine, and glycemia were significantly higher in nonsurvivors than survivors (*P*<.001). Nonsurvivors had a significantly lower lymphocyte and eosinophil percentage and red blood cell count as well as lower hemoglobin, mean corpuscular hemoglobin concentration (MCHC), and hematocrit values *(P*<*.*001). Furthermore, nonsurvivors showed significantly higher levels of inflammatory biomarkers such as neutrophil count, C-reactive protein, and neutrophil-to-lymphocyte ratio (*P*<.001). Other differences in laboratory findings among the two groups are summarized in Table 2.

**Table 2:** Laboratory findings upon admission for the overall study sample and a comparison of survivors and nonsurvivors.

Laboratory parameter	All patients (N=852), mean (SD)	Patients discharged alive (n=559), mean (SD)	Deceased patients (n=293), mean (SD)	*P* value^a^
Glucose (mg/dl)	145 (66)	137 (59)	159 (76)	*.001*
Urea (mg/dl)	57 (40)	47 (24)	76 (54)	*.001*
Creatinine (mg/dl)	1.24 (0.90)	1.06 (0.54)	1.59 (1.27)	*.001*
Sodium (mEq/l)	137 (8)	137 (8)	137 (7)	.24
Potassium (mEq/l)	4.17 (0.55)	4.14 (0.49)	4.24 (0.65)	*.04*
Chloride (mEq/l)	99.26 (7.21)	98.84 (7.19)	100.05 (7.17)	*.02*
Total bilirubin (mg/dl)	0.75 (0.48)	0.72 (0.35)	0.82 (0.66)	*.02*
Direct bilirubin (mg/dl)	0.22 (0.60)	0.21 (0.69)	0.25 (0.37)	.31
AST^b^ (U/L)	61 (84)	53 (37)	79 (136)	*.004*
ALT^c^ (U/L)	48 (70)	47 (44)	48 (103)	.90
LDH^d^ (U/L)	430 (220)	391 (160)	509 (292)	*.001*
Creatine kinase (U/L)	300 (637)	231 (387)	429 (932)	*.001*
Amylase (U/L)	73 (48)	69 (37)	80 (63)	*.01*
Lipase (U/L)	47 (72)	43 (46)	56 (105)	.06
Serum cholinesterase (U/L)	6275 (1858)	6674 (1763)	5576 (1812)	*.001*
WBC^e^ × 10^3^/µl	8.12 (4.68)	7.86 (4.72)	8.63 (4.56)	*.02*
RBC^f^ × 10^6^/µl	4.69 (0.72)	4.79 (0.68)	4.51 (0.77)	*.001*
Hemoglobin (g/dl)	13.59 (1.91)	13.83 (1.72)	13.14 (2.16)	*.001*
Hematocrit (%)	41.84 (5.70)	42.37 (5.34)	40.83 (6.22)	*.001*
MCV^g^ (fl)	89.74 (6.66)	89.18 (5.62)	90.80 (8.19)	*.003*
MCH^h^ (pg)	29.13 (2.38)	29.05 (2.12)	29.28 (2.80)	.23
MCHC^i^ (g/dl)	32.43 (1.36)	32.56 (1.15)	32.17 (1.66)	*.001*
Platelets × 10^3^/µl	217.75 (117.90)	221.08 (127.10)	211.41 (97.72)	.22
RDW^j^ (%)	13.65 (1.65)	13.27 (0.27)	14.29 (1.99)	*.001*
Neutrophils (%)	77.45 (11.57)	75.81 (11.75)	80.56 (10.55)	*.001*
Lymphocytes (%)	15.17 (9.20)	16.48 (9.45)	12.67 (8.15)	*.001*
Monocytes (%)	6.89 (4.30)	7.16 (4.01)	6.36 (4.76)	*.02*
Eosinophils (%)	0.32 (0.91)	0.38 (1.05)	0.20 (0.54)	*.001*
Lymphocytes × 10^3^/µl	1.09 (0.99)	1.15 (0.94)	0.98 (1.09)	*.03*
Monocytes × 10^3^/µl	0.51 (0.41)	0.52 (0.35)	0.51 (0.51)	.77
Eosinophils × 10^3^/µl	0.02 (0.07)	0.03 (0.08)	0.02 (0.05)	*.04*
Neutrophils × 10^3^/µl	6.41 (3.72)	6.05 (3.41)	7.11 (4.15)	*.001*
PT^k^ (seconds)	15.84 (8.38)	15.07 (5.83)	17.03 (11.11)	*.02*
Prothrombin activity (%)	68.40 (15.96)	69.86 (14.38)	66.27 (17.82)	*.009*
INR^l^	1.40 (0.76)	1.34 (0.65)	1.51 (0.93)	*.01*
PTT^m^ (seconds)	31.70 (5.74)	31.32 (4.48)	32.29 (7.22)	.08
PTT ratio	1.02 (0.19)	1.00 (0.14)	1.04 (0.25)	.06
C-reactive protein (mg/dl)	11.19 (8.55)	9.85 (7.88)	13.74 (9.17)	*.001*
NLR^n^	7.99 (6.74)	6.78 (5.04)	10.27 (8.68)	*.001*

^a^*P* value refers to either the Student *t* test or the chi-square test. Italicized values are significant.

^b^AST: aspartate aminotransferase.

^c^ALT: alanine aminotransferase.

^d^LDH: lactate dehydrogenase.

^e^WBC: white blood cell count.

^f^RBC: red blood cell count.

^g^MCV: mean corpuscular volume.

^h^MCH: mean corpuscular hemoglobin.

^i^MCHC: mean corpuscular hemoglobin concentration.

^j^RDW: red cell distribution width.

^k^PT: prothrombin time.

^l^INR: international normalized ratio.

^m^PTT: partial thromboplastin time.

^n^NLR: neutrophil-to-lymphocyte ratio.

### Significant Predictors and the Piacenza Score

Using the forward-search algorithm, the following 6 most important predictors at hospital admission were identified and used to compute the Piacenza score: age, MCHC, PaO_2_/FiO_2_ ratio, temperature, previous cerebrovascular stroke, and gender.

**Figure 1:**
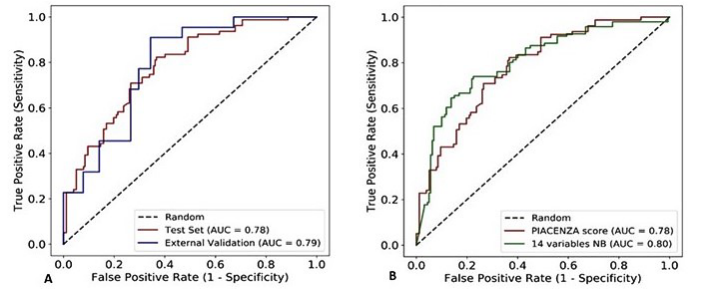
(A) Receiver operating characteristic (ROC) curves obtained by evaluating the Piacenza score (red curve) on the test cohort and on the external validation cohort. (B) ROC curves obtained by evaluating the Piacenza score (red curve) and the naïve Bayes (NB) model trained with 14 manually chosen features (green curve). AUC: area under the ROC curve.

The median of the ROC curve over 100 test cohorts (generated through bootstrapping) is reported in Figure 1. The corresponding median of the AUC is equal to 0.78 (95% CI 0.74-0.84) with a sensitivity of 94% and specificity of 37%. The NPV of the Piacenza score was 93% with a PPV of 40% (Table 3).

The calibration plot of the Piacenza score over the range of risk showed a Brier score of 0.19. The risk deciles are grouped into three levels: low risk (first to fifth deciles), intermediate risk (sixth to eighth deciles), and high risk (ninth and tenth deciles). A gradual and progressive increase in absolute event rates was observed across risk classes for all the Piacenza scores (death: 14% [18/125] in low-risk deciles vs 36% [27/75] in intermediate-risk deciles vs 66% [33/50] in high-risk deciles).

**Table 3:** Negative predictive value (NPV), positive predictive value (PPV; or precision), accuracy, sensitivity (or recall), specificity, F1 score, and F2 score for all scores. These metrics have been calculated for a specific threshold value on the final risk score probability chosen by maximizing the F2 score, the reason being that F2 privileges a high recall and therefore a broader confidence for correctly identifying patients at risk.

Scores	Threshold	NPV	PPV	Accuracy	Sensitivity	Specificity	F1 score	F2 score
Piacenza score	0.16	0.93	0.40	0.55	0.94	0.37	0.56	0.74
Piacenza score–external validation	0.16	0.97	0.37	0.57	0.95	0.44	0.53	0.72
Naïve Bayes model trained with 14 manually chosen features	0.04	0.88	0.54	0.67	0.88	0.55	0.67	0.78
4C mortality score	0.12	0.98	0.39	0.53	0.99	0.34	0.56	0.76

**Figure 2:**
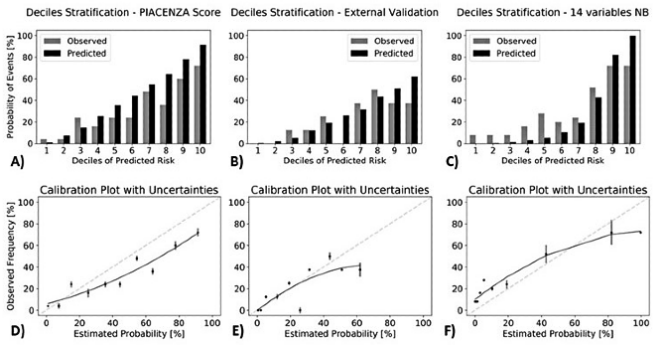
Risk of observed death according to deciles of event probability based on the Piacenza score (A), the Piacenza score on the external validation data set (B), and the naïve Bayes (NB) model trained with 14 manually chosen features (C). For every single case, the corresponding calibration plots with standard deviations calculated over the deciles are also shown below each respective graph (D, E, and F).

From the computed calibration plot, we can observe that the mortality risk is underestimated only in the first few deciles, while in the higher deciles the risk is slightly overestimated (Figure 2A-D).

**Figure 3:**
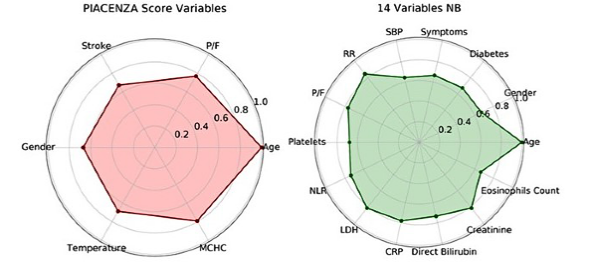
Radar plot for the 6 Piacenza score predictors of death and for the 14 manually chosen features, showing their relative importance. Feature importance is scaled with respect to the most important feature. NB: naïve Bayes, MCHC: mean corpuscular hemoglobin concentration, CRP: C-reactive protein, LDH: lactate dehydrogenase, NLR: neutrophil-to-lymphocyte ratio, P/F: PaO2/FiO2, RR: respiratory rate, SBP: systolic blood pressure.

Regarding the relative importance of each features independent from the others, age was the most important feature to predict death followed by MCHC, PaO_2_/FiO_2_ ratio, previous cerebrovascular stroke, gender, and temperature (Figure 3).

### External Validation

The corresponding median of the AUC in the external validation cohort was 0.79 (95% CI 0.68-0.89) with a Brier score of 0.16 (Figure 1A), a sensitivity of 95%, and a specificity of 44% (Table 3).

The calibration plot is reported in Figure 2B and showed again a gradual and progressive increase in absolute event rates across risk classes (death: 10% [4/40] in low-risk deciles vs 29% [7/24] in intermediate-risk deciles vs 38% [6/16] in high-risk deciles).

### Comparison With the 4C Mortality Score and the Naïve Bayes Model Using Manually Chosen Features

**Figure 4:**
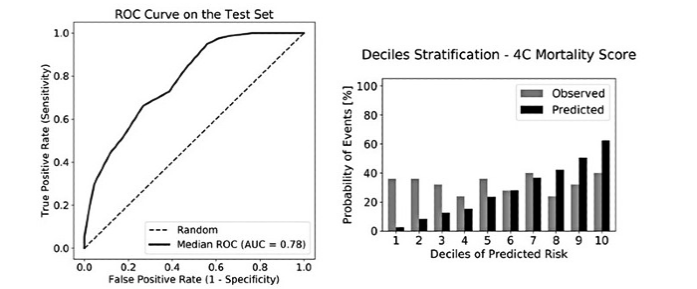
Performance of the 4C mortality score (both in terms of discrimination and calibration abilities) calculated on the test cohort. ROC: receiver operating characteristic, AUC: area under the ROC curve.

The median of the AUC was 0.78 (95% CI 0.73-0.83) with a sensitivity of 99% and specificity of 34% for the 4C score when evaluated on the test cohort. The corresponding Brier score was equal to 0.26 (Figure 4). The naïve Bayes model with 14 features chosen manually based on clinician experience achieved an AUC of 0.80 (95% CI 0.75-0.86) with a sensitivity of 88%, a specificity of 55%, and a Brier score of 0.17 (Figure 1B). The detailed performance metrics of both scores are reported in Table 3. The relative importance of the selected 14 features of the naïve Bayes model is shown on the radar plot in Figure 3.

The observed mortality increased gradually and progressively for the naïve Bayes model with manually chosen features—death: 14% (17/125) in low-risk deciles vs 32% (14/75) in intermediate-risk deciles vs 72% (36/50) in high-risk deciles. This was not observed for the 4C score—death: 33% (41/125) in low-risk deciles vs 31% (23/75) in intermediate-risk deciles vs 36% (18/50) in high-risk deciles. Both scores achieved a satisfactory patient stratification only in the last three deciles whereas the 4C mortality score overestimated the prediction in the high-risk deciles and underestimated it in the low-risk ones (Figures 2C-4).

## Discussion

### Principal Findings

In this study, we developed and validated a machine learning–based risk score—the Piacenza score—to predict mortality risk among hospitalized patients with COVID-19 pneumonia. This score is based on only 6 variables that are readily available at hospital admission.

Satisfactory performance, measured in terms of AUCs in both the testing and external validation cohorts, was achieved with excellent patient stratification. More specifically, the Piacenza score showed a higher sensitivity with a lower specificity. Likewise, it underestimated the mortality risk in the first three risk deciles; slight overestimation occurred in the other deciles. This behavior is acceptable and preferred in an acute setting since the score has been designed as a screening predictive tool capable of correctly identifying patients at low risk from those at high risk of mortality.

In crowded hospitals, and with shortages of medical resources, this simple model can help to quickly prioritize patients: if the patient’s estimated risk is low, the clinician may choose to monitor the patient, whereas a high-risk estimate might support aggressive treatment or admission to the ICU. Data from China, Europe, and the United States reported a hospitalization rate of 20% to 31%, an ICU admission rates from 17% to 35%, and an in-hospital mortality rate between 15% and 40% [13]. In our study, the in-hospital 30-day mortality rate was 34% with lower survival rates for older patients with pre-existing comorbidities and with clinical signs and symptoms suggesting respiratory failure at hospital admission. In line with previous findings, we found that the most common laboratory abnormalities among patients who died were related to the inflammatory process, renal and liver damage, and procoagulation status [14,15].

In the presence of a large number of patients requiring intensive care and threatening to overwhelm health care systems around the world, several models to predict survival and guide clinical decisions in COVID-19 pneumonia were developed [16]. However, many of these models have been found to have a high risk of bias, which could reflect their development based on a small study population with high risk of overfitting and poor generalization properties to new cohorts, and without clear details of model derivation and testing [6].

The recent spread of artificial intelligence has brought novel ways to combat current global pandemics by collecting and analyzing large amounts of data, identifying trends, stratifying patients on the basis of risk, and proposing solutions at the population level instead of at the single individual level [17,18].

### Comparison With Other Risk Stratification Scores

During the COVID-19 pandemic, machine learning approaches have been used to predict the outbreak, to diagnose the disease, to analyze chest x-ray and CT (computed tomography) scan images, and more recently to predict mortality or progression risk to severe respiratory failure [19,20].

Yuan and colleagues [21] developed a simple prognostic risk score based on a logistic regression classifier that included 3 laboratory markers: lactate dehydrogenase, high-sensitivity C-reactive protein, and lymphocyte percentage. This score was developed from a cohort of 1479 patients and externally validated in 2 independent cohorts, reaching an accuracy of 95% in predicting the risk of mortality. However, the model comprised only Chinese patients during the early stages of the outbreak and, more importantly, it seems to have a significant selection bias as it did not include patients with mild and moderate disease at admission [21].

The 4C mortality score, developed and validated by the International Severe Acute Respiratory and Emerging Infections Consortium, based on 8 clinical and laboratory variables, achieved an AUC of 0.78 in predicting mortality. It is easy to use and has a pragmatic design. In fact, to calculate the score, no external tool or complex mathematical equation is required, and results can be immediately retrieved at the bedside [5]. However, due to the rapidly evolving characteristics of the virus and its impact on the population, the score should be continuously updated. For example, the 4C score did not include patients from the second wave of the pandemic. At the same time, if a broad range of individuals are included, the score may become unsuitable for more specific clinical scenarios, such as patients affected by severe pneumonia.

The performance of our model is comparable with the 4C mortality score applied to the test cohort used in this paper. However, we remark that the 4C mortality score was derived based on a population of 35,000 patients, while the naïve model providing the Piacenza score was trained using information coming only from 852 patients. This is indicative of the high representativeness of the training cohort considered in our study. Furthermore, although there is a similar discriminative power between the 4C score and the Piacenza score, the latter score showed better performance in stratifying patients according to their mortality risk, which is of paramount importance in selecting the appropriate treatment and for resource allocations. We also externally tested our score, achieving good performance and confirming that our data-driven model is robust despite its reliance on variables deemed relevant in this context without actually knowing their semantics.

### Characteristics of the Piacenza Score

The Piacenza score contains parameters reflecting patient demographics, comorbidity, and physiology at hospital admission. It shares some characteristics with the 4C score such as age, gender, comorbidities, and PaO_2_/FiO_2_ but also includes unexplored features like temperature and MCHC deriving from a substantially different selection of variables. Unlike traditional scores based on logistic regression analysis mixed with a knowledge-driven approach where a score is assigned by an expert to each of the limited number of selected variables, the proposed predictive model is purely data driven and is not affected by a clinically oriented, potentially biased choice of variables [22].

The Piacenza score is highly customizable and can be adapted as more information becomes available on disease progression and the impact of interventions like vaccines and new pharmacological treatments. In fact, the naïve Bayes algorithm, during its learning phase, generates a summary of the data set where each variable is associated with the outcome in terms of a probabilistic dependence. This summary describes the distribution of the current data set and can be quickly and easily updated when a new observation is available, adapting itself to changes within the population. Likewise, if new data are available, they can be used to train a new version of the Piacenza score and study the possible fingerprints of COVID-19 variants.

The Piacenza score is thus highly flexible; if the some of the required variables are missing, the model can be retrained and the physician can still receive a customized result (associated with the best possible accuracy with respect to the variables provided). The retraining process can take up to 10 hours, depending on the number of features inserted. However, depending on future requests, codes can be easily optimized and run on more powerful hardware.

An example of a personalized model different from the Piacenza score is the naïve Bayes model trained with 14 manually chosen features, which showed a predictive power comparable to that of the Piacenza score. Other models differ in performance; however, as demonstrated, the variables age and PaO_2_/FiO_2_ ratio have the biggest contribution to the predictive power of the model. Therefore, starting with age and the PaO_2_/FiO_2_ ratio and adding more variables will lead to predictive performances similar to that of the Piacenza score, which represents the best combination for stratifying patients and predicting mortality.

Finally, our score’s predictors were not chosen a priori (like, for example, the 4C mortality score) but as the product of a machine learning–based optimization technique, which considers the smallest possible subset of leading predictors associated with the best possible performance.

### The Piacenza Score Beyond the COVID-19 Pandemic

The approach proposed in our paper is suitable for risk stratification and mortality assessment of other conditions as well, such as heart failure (HF), which constitutes a growing public health issue. In fact, although machine learning has made significant contributions to health care in just a few years, little evidence exists on the role of machine learning in predicting mortality in patients with HF and in general with cardiovascular diseases. In this context, several researchers have developed prognostic risk scores for HF such as the Seattle Heart Failure Model and the Meta-Analysis Global Group in Chronic Heart Failure [23,24]. However, these models do not necessarily predict mortality in patients with HF at the individual level and do not present the same flexibility as the Piacenza score. When dealing with cardiovascular diseases, the flexibility of the scores is of crucial importance due to the continuous and rapid changes in therapeutic strategies; this makes the above-mentioned scores less useful or not reliable in clinical practice.

### Limitations

This study has room for further improvement, which is left for future work. First, given that the proposed machine learning method is purely data driven, our model may vary if a different data set is used. As more data become available, the model can be refined and performance of the Piacenza score can further increase. To this aim, we are currently looking forward to subsequent large-sample and multicentered studies. Second, the forward-selection algorithm (used to select the Piacenza score predictors and most importantly to personalize the Piacenza score on any other subset of features) may be an expensive option to be considered and may surely be optimized in further versions of the code. Finally, new variables such as d-dimer and troponin, currently not available, but which are known to be associated with a higher mortality risk in cases of COVID-19 pneumonia may be included in future analyses.

### Conclusion

In conclusion, we have developed and validated robust machine learning models, which could be used to predict the prognosis of patients with COVID-19. The Piacenza score has several advantages: first, it relies on objective clinical and laboratory measurements not affected by human interpretation; second, it was tested and validated in patients belonging to the second wave of the pandemic; third, it is automatically generated through a combination of variables widely available at hospital admission and can be calculated through a user-friendly web interface; and finally, as opposed to traditional epidemiological predictive models, the Piacenza score has the added advantage of adaptive learning, trend-based recalibration, and flexibility.

## Abbreviations

AUC: area under the receiver operating characteristic curve
CSV: comma-separated values
CT: computed tomography
EHR: electronic health record
HF: heart failure
ICU: intensive care unit
MCHC: mean corpuscular hemoglobin concentration
NPV: negative predictive value
PPV: positive predictive value
ROC: receiver operating characteristic
RT-PCR: reverse transcriptase–polymerase chain reaction
